# Pediatric COVID-19 Involving Complicated Sinusitis With Intracranial Extension and Lemierre Syndrome: A Case Report

**DOI:** 10.7759/cureus.34960

**Published:** 2023-02-14

**Authors:** Rohit Nallani, Michael E Price, Srivats S Narayanan, Meghan Tracy, Jill Arganbright

**Affiliations:** 1 Otolaryngology - Head and Neck Surgery, University of Kansas Medical Center, Kansas City, USA; 2 School of Medicine, University of Missouri at Kansas City, Kansas City, USA; 3 Otolaryngology, Children's Mercy Hospital, Kansas City, USA

**Keywords:** complicated sinusitis, paranasal sinusitis, lemierre's syn, coronavirus disease, sars-cov-2 (severe acute respiratory syndrome coronavirus -2), covid-19

## Abstract

Pediatric coronavirus disease 2019 (COVID-19) has been associated with various complications including chronic respiratory disease and multisystem inflammatory syndrome. There are a few reported cases of complicated sinusitis following pediatric COVID-19 infection. We present a patient with recent COVID-19 who developed complicated sinusitis with intracranial extension and Lemierre syndrome. A 16-year-old female with a history of COVID-19 diagnosis 17 days prior presented with worsening head and neck symptoms. Physical examination demonstrated left proptosis, cranial nerve (CN) VI palsy, and limited neck range of motion. Imaging demonstrated bilateral sinus disease, a 3.3 × 2 × 3-centimeter sellar/clival abscess, bilateral cavernous sinus thrombosis, and thrombosis of bilateral internal jugular veins. Urgent endoscopic sinus surgery was performed, and long-term intravenous antibiotics and anticoagulation were initiated with improvement in symptoms over three weeks. Providers caring for patients with COVID-19 should keep complicated sinusitis and Lemierre syndrome in their differential. Further study of COVID-19 pathophysiology in the sinonasal mucosa is needed.

## Introduction

The coronavirus disease 2019 (COVID-19) global pandemic caused by the novel severe acute respiratory syndrome coronavirus 2 (SARS-CoV-2) has been found to impact many organ systems. While respiratory symptoms of cough, chest pain, and shortness of breath are classic presenting features, otolaryngologic symptoms including sore throat, nasal congestion/obstruction, and taste/smell disturbances were quickly discovered to be prevalent [1,2]. These same ear-nose-and-throat symptoms have been shown to occur greater than 20% of the time in children with COVID-19 [3]. Other uncommon otolaryngologic complications such as cranial nerve (CN) palsies have also been reported following COVID-19 [4].

Pediatric patients infected with SARS-CoV-2, while typically having milder symptoms [5], can develop life-threatening bacterial complications, chronic respiratory disease, and multisystem inflammatory syndrome (MIS-C). MIS-C, a syndrome causing inflammation of multiple organ systems, typically affects children above age five and adolescents, presents 1-2 weeks after COVID-19 symptoms, and disproportionally affects Black and Hispanic children [6]. While the precise pathophysiology of MIS-C is unclear, hypotheses support an intense cytokine storm post infection resulting in systemic inflammation, shock, and subsequent multiorgan dysfunction, particularly of the heart and gastrointestinal tract [7]. Given the variable findings and similarities to other pediatric systemic illnesses, diagnosis is challenging. There is also little literature on how COVID-19 and subsequent MIS-C may predispose children to otolaryngologic complications.

A few cases of complicated sinusitis have been documented following SARS-CoV-2 infection [8-10]. Overall, complicated sinusitis is more common in the pediatric population, hypothesized to be a sequela of the anatomic differences in the sinonasal anatomy [11]. Moreover, recent evidence suggests that SARS-CoV-2 directly induces mucociliary dysfunction. We present the case of an adolescent patient with recent COVID-19 who developed complicated sinusitis with intracranial extension and thrombosis of the bilateral internal jugular veins with *Fusobacterium necrophorum* bacteremia, or Lemierre syndrome. Informed consent was obtained to report the case and present the de-identified images and details.

This article was previously presented as a meeting abstract/poster at the American Society of Pediatric Otolaryngology meeting at the Combined Otolaryngology Sections Meeting during April 27-May 1st, 2022 as well as at Children's Mercy Research Day in May 2022.

## Case presentation

A 16-year-old Black female, with a history only of seasonal allergies, presented to the Emergency Room following a COVID-19 diagnosis 17 days prior. The patient complained of progressing and worsening symptoms, including headache, fatigue, photophobia, visual changes, and neck stiffness. Physical examination demonstrated left eye proptosis, CN VI palsy, and limited neck range of motion. The patient was febrile to 104° Fahrenheit, and hypotensive, and laboratory workup revealed an elevated white blood count of 12.7, c-reactive protein (CRP), erythrocyte sedimentation rate, creatine kinase, lactate dehydrogenase, fibrinogen, D-dimer, and B-type natriuretic peptide (Table 1). Anemia and thrombocytopenia were also noted. Initial concerns for MIS-C prompted a transfer to a tertiary-care medical center. Neurology and Infectious Disease teams were immediately engaged.

**Table 1:** Summary of laboratory findings

Lab/measurement	Result	Reference range
White blood count	12.7	4.5-11.0 x 10^3^/mcL
Hemoglobin	9.8	12.0-16.0 g/dL
Hematocrit	29.3	36.0-46.0%
Platelets	91	150-450 x 10^3^/mcL
C-reactive protein	25	0.0-1.0 mg/dL
Erythrocyte sedimentation rate	119	0.0-10 mm/h
Creatine kinase	2,364	45-230 units/L
NT-pro brain natriuretic peptide	455	0.0-125.0 pg/mL
Lactate dehydrogenase	313	120-290 units/L
Fibrinogen	616	164-382 mg/dL
D-dimer	2.53	0.0-0.49 mcg/mL
Albumin	1.8	3.0-5.1 g/dL

Imaging

**Figure 1:**
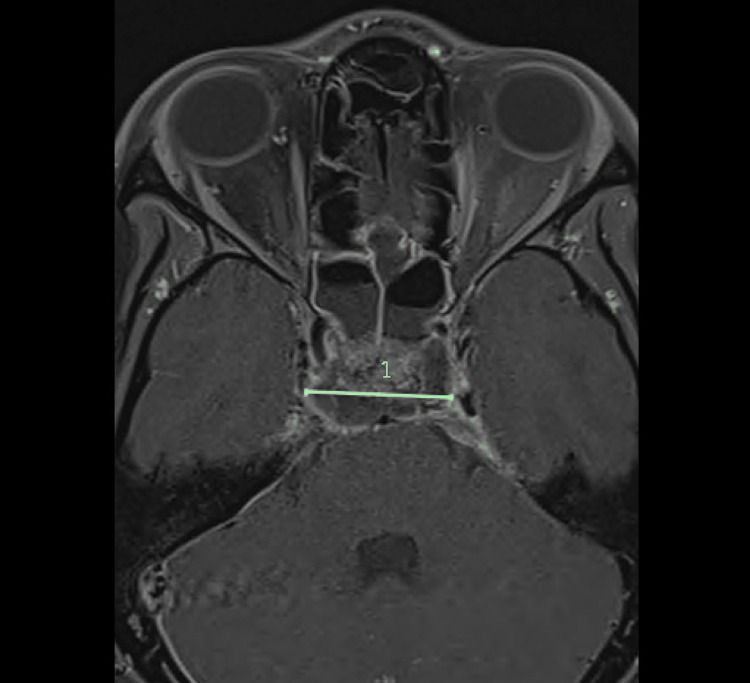
MRI of the brain/orbit with contrast, axial cut, 3.3 cm transverse abscess (scale) at the dorsal aspect of the sella and clivus with involvement of the cavernous sinus bilaterally MRI, magnetic resonance imaging.

**Figure 2:**
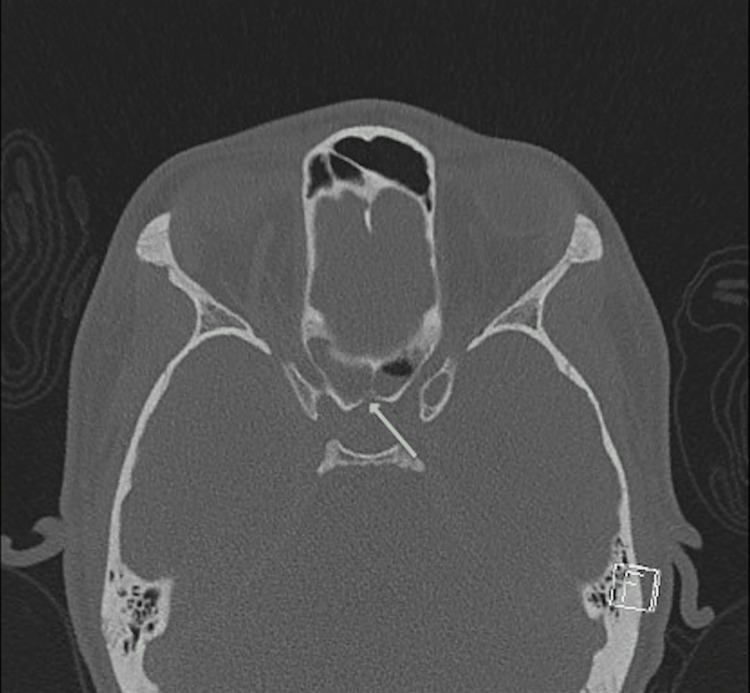
CT scan of sinus with contrast, axial cut, ethmoid and sphenoid sinus disease with bony dehiscence along posterior wall of right sphenoid sinus/anterior sella (arrow) CT, computed tomography.

**Figure 3:**
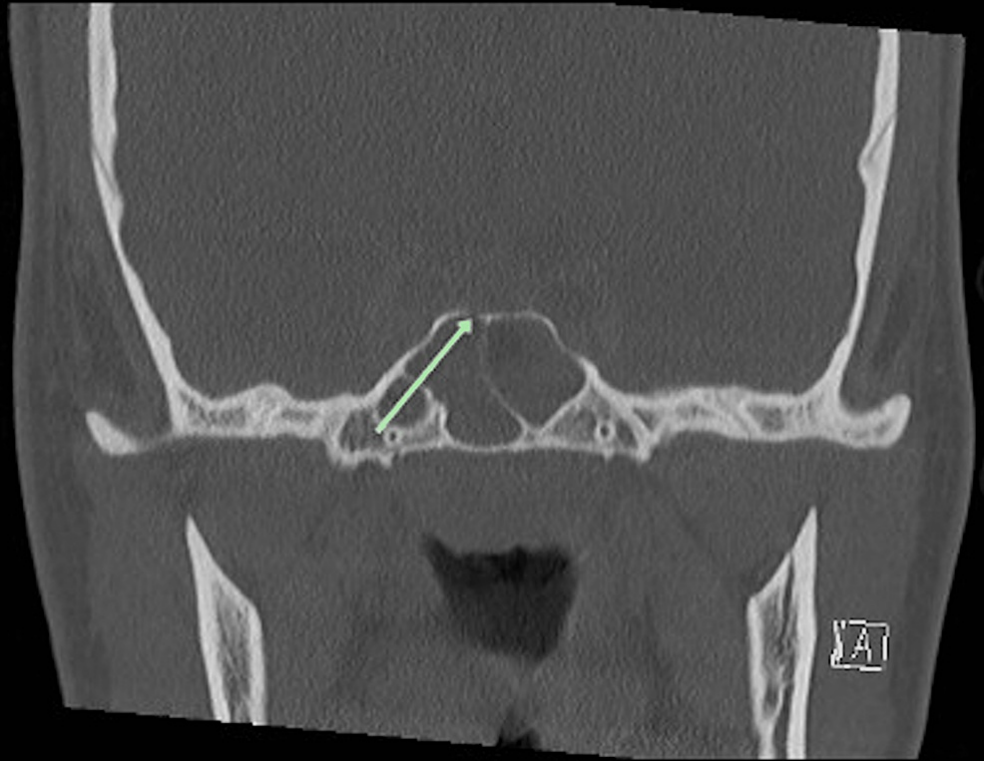
CT of the sinus with contrast, coronal cut, sphenoid sinus disease with bony dehiscence along superior right sphenoid sinus (arrow) CT, computed tomography.

**Figure 4:**
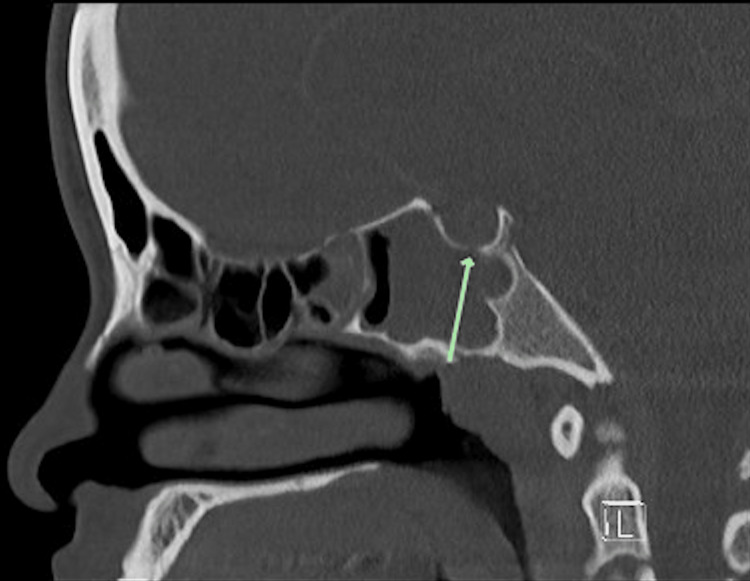
CT of the sinus with contrast, sagittal cut, posterior ethmoid and sphenoid sinus disease with bony dehiscence along superior wall of sphenoid sinus/anterior sella (arrow) CT, computed tomography.

A magnetic resonance imaging (MRI) scan of the brain with contrast demonstrated signs of meningitis and a 3.3 × 2.0 × 3.0-centimeter intracranial abscess extending along the dorsal aspect of the sella and clivus with extension anteriorly to involve the cavernous sinus bilaterally, worse on the left, along with bilateral cavernous sinus thrombosis (Figure 1). A computed tomography (CT) scan of the sinuses demonstrated severe mucosal thickening and opacification of the bilateral sphenoid and right maxillary sinuses with bony dehiscence of the posterior sphenoid sinus wall and right maxillary sinus wall adjacent to the sella (Figures 2-4). Additionally, a CT scan of the neck and chest showed thrombosis of the bilateral internal jugular veins near the skull base, along with multiple bilateral cavitary lung lesions and pleural effusions, concerning embolic disease, and consistent with Lemierre syndrome. Surgical consultations were requested for both otolaryngology and neurosurgery, and the patient was transferred to the pediatric intensive care unit.

Treatment

**Figure 5:**
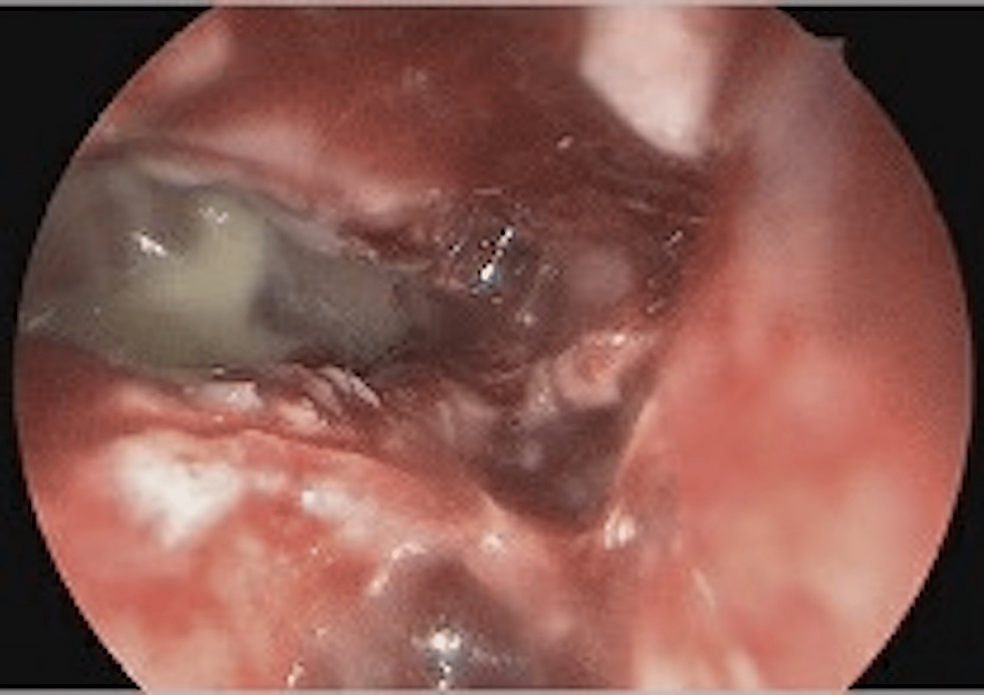
Purulent secretions and edematous mucosa of left sphenoid sinus

The patient underwent urgent endoscopic sinus surgery, which included bilateral sphenoidotomies and right maxillary antrostomy. Neurosurgical intervention was not immediately pursued secondary to the morbidity associated with surgical drainage of her abscess (clivus/sella and cavernous sinus location). There were no intraoperative complications. The nasal mucosa was edematous, and copious purulence was noted in the sinonasal cavities (Figure 5). Sinus bacterial cultures grew *Prevotella melaninogenica, Cutibacterium acnes, Staphylococcus epidermidis, and *Methicillin-sensitive* Staphylococcus aureus (MSSA)*. Blood cultures were also positive for *Fusobacterium necrophorum*, which provided further evidence for Lemierre syndrome. Following surgery, the patient was started on a standard nasal hygiene regimen of twice-daily saline irrigations and intranasal corticosteroids. The patient was also treated with culture-targeted antibiotics for a total of six weeks (Table 2). Anticoagulation was also initiated one day after surgical intervention and continued for six weeks (Table 2). Given persistent fevers and worsening of left pleural effusion, a chest tube was placed on hospital day 7. A repeat MRI on hospital day 7 demonstrated the progression of sinus disease and intracranial inflammation, and the patient subsequently underwent a repeat sinus washout procedure on hospital day 9, with edematous mucosa and mucus visualized without purulence. Neurosurgical intervention was never performed due to the location of the intracranial abscess. The patient’s headaches, vision changes, and energy levels slowly improved, the chest tube was removed on hospital day 12, and a repeat MRI on postoperative day 20 showed improvement of the sinus disease, cavernous sinus thrombosis, and intracranial abscess. CRP on discharge was 0.5 mg/dL. The patient was discharged home on hospital day 21. 

**Table 2:** Antibiotic and anticoagulation durations

Antibiotic	Duration
Cefepime	2 weeks (hospital days 0-2, hospital days 7-21)
Metronidazole	7 weeks (hospital days 1-21, 4 weeks following discharge)
Vancomycin	5 days (hospital days 0-5)
Ceftriaxone	3 days (hospital days 2-5)
Cefazolin	2 days (hospital days 5-7)
Linezolid	4 weeks (started on discharge)
Anticoagulation	
Heparin	1 day (hospital days 2-3)
Bivalirudin	9 days (hospital days 3-12)
Rivaroxaban	5 weeks (hospital day 12 to 4 weeks following discharge)

Follow-up

The patient did not present to her scheduled otolaryngology or neurosurgery clinic visits following discharge. Approximately one month after discharge, Infectious Disease and Hematology noted the resolution of the infection with the only persistent symptom being left CN VI palsy, suspected secondary to inflammation injury. However, the patient has had persistent, but improving, blurry vision six months later.

## Discussion

While SARS-CoV-2 has an affinity for the respiratory epithelium and can manifest with many otolaryngologic symptoms, progression to complicated otolaryngologic disease is rare. While unclear if infection with SARS-CoV-2 was the precipitating event for the development of complicated sinusitis and Lemierre syndrome in this patient or a coincidental finding, other published reports offer support for COVID-19 possibly predisposing patients to severe illness in adolescents [6,8,10,12]. Regardless, this is a rare case of complicated sinusitis with intracranial abscess and Lemierre syndrome in a pediatric patient following SARS-CoV-2 infection. Early intervention and multispecialty management allowed for adequate treatment for this patient.

Recent reports by Arteaga et al. [8] and Turbin et al. [10] have documented similar cases of complicated sinusitis in the setting of COVID-19 in adolescents. In the former, a 14-year-old Black male was diagnosed with MIS-C and had rapid progression of infection following admission to an intracranial abscess, dural venous sinus thrombosis, and orbital abscesses. Following neurological and otolaryngological surgical intervention and treatment with broad-spectrum antibiotics, he was eventually discharged on hospital day 20. He did require a sinus washout on day 8 following the development of periorbital and frontal swelling. In the latter case report, a 12-year-old Egyptian male and a 15-year-old Black male presented with complicated sinusitis and tested positive for SARS-CoV-2. The 12-year-old required incision and drainage of a subperiosteal abscess and no sinus surgery. The 15-year-old required sinus surgery for the treatment of sinusitis, meningitis, and an epidural abscess. Both patients were also treated with extended courses of intravenous antibiotics. These cases highlight the importance of surgery in addition to antibiotics in managing these patients. Another more common cause of intracranial thrombosis in children is complicated acute otitis media, which also often requires surgery and intravenous antibiotics [13].

The progression to Lemierre syndrome is a unique finding in this case. Lemierre syndrome is defined as a systemic infection with septic thrombophlebitis of the internal jugular vein with septic emboli affecting multiple organs, most commonly the brain, bone, or lungs. Blood cultures positive for *Fusobacterium necrophorum* in the context of these symptoms are pathognomonic for the disease, as seen in this patient. Lemierre syndrome is most common in adolescents and young adults. A report by de Marcellus et al. is the only published case of Lemierre syndrome following SARS-CoV-2 infection [12]. Given documented beta-lactam resistance of Fusobacterium, treatment with antibiotics with anaerobic activity and beta-lactamase resistance is often sufficient [14]. Our patient was treated with multiple antibiotics for sinus and systemic bacterial infections, with the longest course consisting of cefepime/linezolid and metronidazole (Table 2).

The role of anticoagulation for Lemierre syndrome is controversial. Arguments for use suggest managing diseases like venous thrombosis where anticoagulation is beneficial [14]. Arguments against cite risks of bleeding and hemorrhagic transformation of septic emboli [14]. In our case, the decision was made to start anticoagulation from the recommendations of infectious disease and hematology specialists and based on the intracranial and pulmonary findings. Symptomatic venous thrombosis and intracranial extension have been offered as indications for anticoagulation in Lemierre syndrome [14]. While heparin was initially started, the patient was eventually switched to bivalirudin due to subtherapeutic levels with prophylactic heparin and extension of pulmonary emboli. de Marcellus et al. [12] reported on an adolescent patient with Lemierre syndrome and COVID-19 who died following a stroke and vasculitis while on prophylactic enoxaparin despite being treated with adequate antibiotics and undergoing sinus surgery. Further study of adequate anticoagulation of pediatric patients in these situations is needed especially given the high morbidity and mortality associated with Lemierre syndrome [12,14].

While our patient did not meet the criteria for a diagnosis of MIS-C due to alternative diagnoses (Lemierre syndrome and complicated sinusitis), her prior SARS-CoV-2 infection cannot be ignored as a precipitating factor for her illness. A study of SARS-CoV-2 has discovered the robust expression of its target receptors - angiotensin-converting enzyme 2 and transmembrane protease serine-2 - in oropharyngeal and sinonasal mucosa, enabling ease of virus entry into human tissue and transmission [15,16]. Interestingly, these receptors are less frequently expressed in children, explaining the overall lower morbidity and mortality in pediatric patients during the pandemic [17]. Severe SARS-CoV-2 infection is likely a result of a dysregulated immune system with increased production of pro-inflammatory cytokines, the heightened presence of neutrophils, and a failure of CD8+ lymphocytes and natural killer cells' defense against the virus [17]. This can eventually lead to MIS-C in children, hypercoagulability, and deadly neurological sequela [5,18]. We hypothesize that this patient’s prior SARS-CoV-2 infection led to an immune breakdown and a susceptibility to concurrent bacterial infection with progression to complicated sinusitis and Lemierre syndrome. Further study of the pathophysiology of SARS-CoV-2 in pediatric patients in targeting the sinonasal mucosa and factors influencing susceptibility, such as allergic and sinonasal disease, to severe bacterial infection is needed.

## Conclusions

This is a presentation of an adolescent patient with recent COVID-19 and suspected MIS-C who developed complicated sinusitis with intracranial extension and thrombosis of the bilateral internal jugular veins with *Fusobacterium necrophorum* bacteremia. Early surgical intervention, long-term intravenous antibiotics, and anticoagulation were important for her recovery. Providers caring for pediatric patients with severe COVID-19 and initial concerns for MIS-C should keep complicated sinusitis and Lemierre syndrome in their differential, especially in patients with sinonasal, neurological, and pulmonary symptoms. Further study of SARS-CoV-2 pathophysiology in the sinonasal mucosa and in patients with the comorbid allergic and sinonasal disease is needed. 
